# Retraction Note: The impact of vitamin A supplementation on thyroid function and insulin sensitivity: implication of deiodinases and phosphoenolpyruvate carboxykinase in male Wistar rats

**DOI:** 10.1007/s00394-024-03483-y

**Published:** 2024-08-28

**Authors:** Samar R. Saleh, Rania Zaki, Radwa Hassan, Mohamed A. El-Kersh, Mohamed M. El-Sayed, Alshimaa A. Abd Elmoneam

**Affiliations:** Biochemistry Department, Faculty of Science, Alexandria University, Alexandria, Egypt

**Retraction Note: European Journal of Nutrition (2022) 61:4091–4105**

10.1007/s00394-022-02945-5

The Editor in Chief has retracted the article. Concerns have been raised regarding a number of figures, specifically:

• Apparent reuse of GADPH controls with apparent rearrangement across Fig. 6 weeks 2, 4 and 6 and Fig. 7 weeks 2, 4 and 6.

• Apparent duplicate bands across Fig. 7 PEPCK weeks 2, 4 and 6.

• Three instances of apparent duplicate bands within each of weeks 2, 4 and 6.

The Editor-in-Chief therefore no longer has confidence in the results and conclusions reported in this article.

